# OctylPhenol (OP) Alone and in Combination with NonylPhenol (NP) Alters the Structure and the Function of Thyroid Gland of the Lizard *Podarcis siculus*

**DOI:** 10.1007/s00244-021-00823-5

**Published:** 2021-03-09

**Authors:** Rosaria Sciarrillo, Mariana Di Lorenzo, Salvatore Valiante, Luigi Rosati, Maria De Falco

**Affiliations:** 1 Department of Science and Technologies, University of Sannio, Benevento, Italy; 2 Department of Biology, University of Naples “Federico II”, Naples, Italy; 3 National Institute of Biostructures and Biosystems (INBB), Rome, Italy; 4 Center for Studies On Bioinspired Agro-Environmental Technology (BAT Center), Portici, Italy

## Abstract

**Abstract:**

Different environmental contaminants disturb the thyroid system at many levels. AlkylPhenols (APs), by-products of microbial degradation of AlkylPhenol Polyethoxylates (APEOs), constitute an important class of Endocrine Disrupting Chemicals (EDCs), the two most often used environmental APs being 4-nonylphenol (4-NP) and 4-tert-octylphenol (4-t-OP). The purpose of the present study was to investigate the effects on the thyroid gland of the bioindicator *Podarcis siculus* of OP alone and in combination with NP. We used radioimmunoassay to determine their effects on plasma 3,3′,5-triiodo-L-thyronine (T_3_), 3,3′,5,5′-L-thyroxine (T_4_), thyroid-stimulating hormone (TSH), and thyrotropin-releasing hormone (TRH) levels in adult male lizards. We also investigated the impacts of AP treatments on hepatic 5′ORD (type II) deiodinase and hepatic content of T_3_ and T_4_. After OP and OP + NP administration, TRH levels increased, whereas TSH, T_3_, and T_4_ levels decreased. Lizards treated with OP and OP + NP had a higher concentration of T_3_ in the liver and 5′ORD (type II) activity, whereas T_4_ concentrations were lower than that observed in the control group. Moreover, histological examination showed that the volume of the thyroid follicles became smaller in treated lizards suggesting that that thyroid follicular epithelial cells were not functionally active following treatment. This data collectively suggest a severe interference with hypothalamus–pituitary–thyroid axis and a systemic imbalance of thyroid hormones.

**Graphic Abstract:**

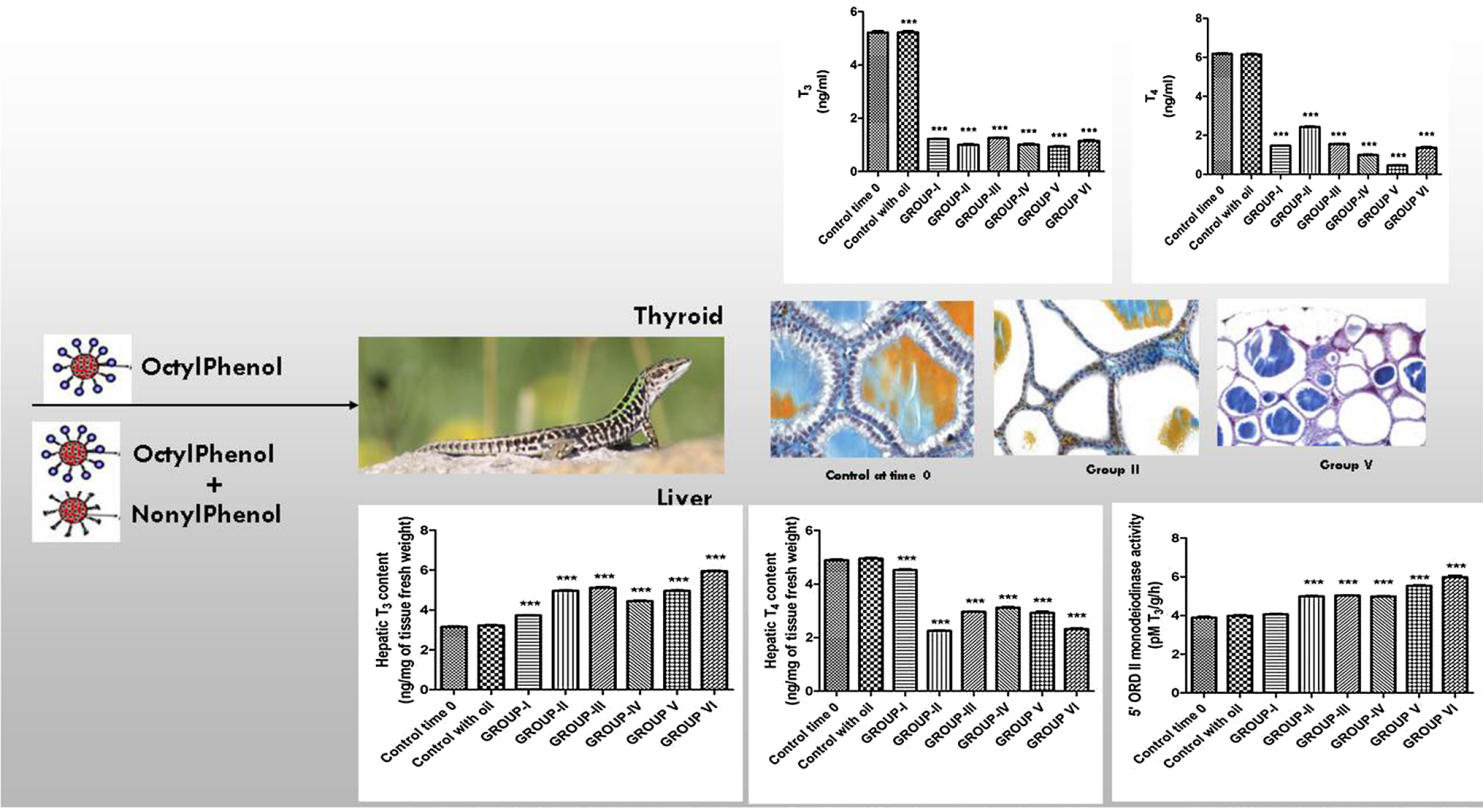

Thyroid is a gland with an endocrine activity and a follicular organization. This gland secretes thyroid hormones (THs), and its secretory activity is regulated by the thyroid-stimulating hormone (TSH) secreted by the anterior pituitary gland, which in turn is regulated by the thyrotropin-releasing hormone (TRH) produced by the hypothalamus. This hormonal crosstalk plays a pivotal role in the regulation of thyroid function (Esposito et al. 2015; Hay et al. 2019). The two main thyroid hormones are 3,3′,5,5′-L-thyroxine (T_4_) and the active 3,3′,5-triiodo-L-thyronine (T_3_) (Esposito et al. 2019; Perna et al. 2018) through which thyroid regulates protein synthesis, metabolism, growth, and rate of function of many other systems present in the body (Ji et al. 2012; Santangelo et al. 2016).

The incidence of thyroid diseases has continuously increased in recent years (Tingi et al. 2016), and it seems to correlate to exposure to several environmental pollutants, such as endocrine-disruptor chemicals (EDCs) (Benedetti et al. 2017; Djordjevic et al. 2020).

EDCs are a broad group of persistent and lipophilic compounds detected in different environmental matrices, able to bioaccumulate in animals and humans, and thus interfering temporarily or permanently with the hormonal signaling pathways in the endocrine system. As a consequence, they adversely affect different organs by binding to hormone receptors or interfering with the production, metabolism, and transfer of hormones and/or modifying gene expression (Ghassabian and Trasande 2018). Literature has extensively described the harmful effects of exposure to different EDCs in different organisms at multiple levels. For instance, the pesticide mancozeb, which has been shown to induce neurodegenerative effects in marine teleosts (Zizza et al. 2017, 2018), is considered to act as an oxidative stressor of both gills and blood (Kubrak et al. 2012) and an inductor of oxidative damage of lipids and proteins in brain, liver, and kidney of fishes (Atamaniuk et al. 2013). Mancozeb also exerts numerous injurious effects related to the function of the thyroid gland (Axelstad et al 2011; Goldner et al 2010). Additionally, several reports suggested a xenoestrogenic activity of alkylphenols (APs), such as Bisphenol A (BPA), NonylPhenol (NP), an OctylPhenol (OP) (Acconcia et al. 2017; Forte et al. 2016, 2019; In et al. 2015; Liu et al. 2017). These substances are largely used in industry to optimize the production of different products, such as herbicides, pesticides, lubricants, plastics, and personal care products (Asimakopoulos et al. 2012; Raecker et al. 2011). Their wide use has determined their detection in many environmentally relevant matrices as wastewaters, surface waters, sediments, and soil (Kung et al. 2018). Moreover, they have been detected in several aquatic species (Staniszewska et al 2017) and in human blood samples, including amniotic fluid, urine, breast milk, fetal cord serum, and placenta (Ademollo et al. 2008; Calafat et al. 2007; Shekhar et al. 2017). APs are effective not only on reproductive system, but also can have neurotoxic effects on organisms (Liu et al. 2019), affect adrenal glands (De Falco et al. 2010, 2014; Di Lorenzo et al. 2020a, 2020b), or inhibit cell proliferation in gastric adenocarcinoma (Manente et al. 2011).

Some interesting evidence highlighted the impact of APs on thyroid. BPA, among its different mode of actions, affects the hypothalamic-pituitary-thyroid axis (HPT) both in vitro and in vivo (Benedetti et al. 2017; Fernandez et al. 2018; Moriyama et al. 2002; Tan et al. 2003; Wetherill et al. 2007; Zoeller et al. 2005).

Few studies investigated the effects of NP and OP on HPT, suggesting that both chemicals are capable of interfering with its function and, consequently, influence basic growth as well as development (Göktekin and Barlas 2008; He et al. 2019; Naderi et al. 2014; Wang et al. 2019; Xi et al 2013).

We have already demonstrated in a previous study that acute exposure to NP affects the function of thyroid glands in adult male lizard *Podarcis siculus* (Sciarrillo et al. 2010). The purpose of the present study was to evaluate the effects on the thyroid gland of the lizard species *Podarcis siculus* of OP, administered alone or combined to NP. *P. siculus* was chosen for this study, because it is a sentinel species for biomonitoring the ecotoxicological impact of EDCs, due to its ecological and life history characteristics, such as its distribution in a variety of habitats, wide geographical range, longevity, site fidelity (i.e., philopatric), and high sensitivity to the effects of contaminants (Verderame et al. 2016a,b).

The effects of OP and OP + NP exposure on T_3,_ T_4_, TSH, and TRH plasma levels in adult lizards were determined by radioimmunoassay. Hepatic T_3_ and T_4_ contents and deiodinase types II (5′ORD2) activity also was investigated to identify the effect of OP and OP + NP on the liver, which is the principal target organ of THs. Besides, we investigated the histological changes of the thyroid glands.

## Materials and Methods

### Compounds

OctylPhenol (OP) and NonylPhenol (NP) were obtained from FLUKA (Sigma-Aldrich Co., St. Louis, MO) (ECHA 2019, n.d.).

### Animals and Housing Conditions

Adult specimens of *Podarcis siculus*, weighing 13–15 g, were live-captured in the neighborhood of Naples in June when their thyroid gland was in full functional activity (Sciarrillo et al. 2000). Lizards were maintained in a soil-filled terrarium containing heather and indoor exposed to natural photoperiod and temperature. They were fed with *Tenebrio molitor* larvae and water dishes were always available in the terraria. Before starting the treatments, an acclimatization period of approximately 15 days was allowed to reverse capture-related stress (Rosati et al. 2020). The experiments were performed in accordance with the ethical provisions imposed by the European Union and permitted by the National Committee of the Italian Ministry of Health on in vivo experimentation.

### Experimental Procedure

*Podarcis siculus* specimens were treated with OP and OP + NP. Compound concentrations administered were established based on preliminary dose–response tests and data (De Falco et al. 2014; Di Lorenzo et al. 2020b; Sciarrillo et al. 2010).

Lizards were divided into eight groups (6 treated and 2 control groups), each consisting of ten animals (5 males and 5 females). OP was used at the concentration of 0.161 µg and NP at 0.172 µg; both compounds were dissolved in 50 µL of corn oil and administered through intraperitoneal injections every 2 days. Lizards were daily inspected for signs of toxicity and death.

*Control Group*: Untreated control lizards were sacrificed after having been housed for 20 days in nonpolluted terraria (time zero controls); an additional control group (group treated with oil) was intraperitoneally injected with 50 µL of corn oil for 22 times. From this group, five lizards were sacrificed 24 h after the last injection and five 15 days after last injection.

*Study Group I*: Lizards were treated with 12 intraperitoneal injections of OP and sacrificed 24 h after the last injection.

*Study Group II*: Lizards were treated with 22 intraperitoneal injections of OP and sacrificed 24 h after the last injection.

*Study Group III*: Lizards were treated with 22 intraperitoneal injections of OP and sacrificed 15 days after the last injection (recovery OP group).

*Study Group IV*: Lizards were treated with 10 intraperitoneal injections of OP + NP and sacrificed 24 h after the last injection.

*Study Group V*: Lizards were treated with 17 intraperitoneal injections of OP + NP and sacrificed 24 h after the last injection.

*Study Group VI*: lizards were treated with seventeen (17) intraperitoneal injections of OP + NP and sacrificed 15 days after the last injection (recovery OP + NP group).

Lizards were anesthetized and sacrificed by decapitation immediately after collection of blood samples the day after the last injection.

### Hormone Assay

Blood samples were collected by intracardiac puncture and put into heparinized tubes. Plasma for hormonal dosages was obtained by centrifuging blood samples for 10 min at 1,500 rpm at 4 °C. TRH and TSH levels were determined by immunoradiometric assay (IRMA) as previously reported by Sciarrillo et al. (2009). T_3_ and T_4_ levels were determined using radioimmunoassay (RIA) (Sciarrillo et al. 2009, 2010).

### Hepatic Thyroid Hormones (T_4_ and T_3_) Content and 5-T4 ORD (type II) Monodeiodinase Activity

Livers were removed and rinsed in a buffer composed by 50 nM of MOPS and 1 mM of EDTA at pH 7.4, homogenized in sucrose buffer (0.25 M sucrose, 5 mM Tris, pH 8.0) using a Pyrex tissue grinder held on ice. Homogenate was then centrifuged at 4 °C and 1,000 rpm for 10 min. Pellet was suspended in sucrose buffer and centrifugated again, whereas supernatant was centrifugated at 4 °C and 12,000 rpm for 5 min. The resulting final supernatant (premicrosomal fraction) was centrifugated in an ultracentrifuge at 4 °C and 78,000 rpm for 90 min. Microsomal pellets were suspended in MOPS and stored at − 80 °C. The content of T_3_ and T_4_ in hepatic tissue was determined by RIA and was expressed as ng/mg of tissue (fresh weight) (Sciarrillo et al. 2000). Thirty microliters of the homogenate was incubated at 12 °C for 20 min with 3 volumes of buffer containing 50 mM of 1–4 dithio-DL-threitol (DTT) and 1 nM radiolabeled T4. Cold ethanol was added to the samples, which were kept at 4 °C overnight, and then centrifugated at 4 °C and 1400 rpm for 20 min. Supernatant was used to determine 5′-T4 ORD type II (ORD II) monodeiodinase activity, expressed as pM T_3_/g (of liver)/h (Sciarrillo et al. 2000).

### Light Microscopy

Animals were anaesthetized by hypothermia and decapitated. Thyroid glands were removed and immediately fixed in Bouin’s fixative and processed for light microscopy (LM) observation. Serially cut paraffin sections (7 µm) were stained with Galgano stain and observed using a Zeiss Axioskop microscope. The height of the follicular cells was measured in 30 cells always using the second section of both normal and treated samples every three slides, using a digital system of image (KS 300).

### Statistical Analysis

Statistical analysis was performed using the GraphPad Prism 8 software. Data obtained were expressed as means ± standard error of mean (SEM). Experimental data of all the groups was tested together for significance using one-way ANOVA, followed by Bonferroni's multiple comparison test. Differences were considered statistically significant when the *p* value was at least *p* < 0.05.

## Results

### Sign of Toxicity and Animal Mortality

Sign of toxicity and mortality of the animal were continuously monitored during the experiment. Lizards from groups treated with OP alone or combined to NP showed evident signs of toxicity and mortality (Table 1). Interestingly, lizards remained in groups on the bottom of the terraria moving very slowly when treated with OP.

**Table 1:** Mortality and signs of toxicity of specimens of *Podarcis sicula* treated with OP and OP + NP mixture (see Materials and Methods section)

Treatments	Dead animals (% of animal mortality)	Signs of toxicity
Control time 0	0	None
Control with oil	0	None
OP treated		
Group I	0	Dyspnea
Group II	2 (10%)	Dyspnea, hind-limb paralysis
Group III	2 (10%)	Dyspnea, hind-limb paralysis
OP + NP treated		
Group IV	4 (20%)	Dyspnea, hind-limb paralysis
Group V	4 (20%)	Dyspnea, hind-limb paralysis
Group VI	4 (20%)	Dyspnea, hind-limb paralysis

The effect of OP seemed to correlate with the duration of treatment. Lizards treated with OP (Study Group I) showed dyspnea after 12 intraperitoneal injections, whereas animals from study Group II and III showed hind-limb paralysis after 22 intraperitoneal injections of OP (Table 1). A similar pattern of toxicity was observed in all the groups of lizards treated with the mixture of OP and NP. A mortality of 20% was observed in lizards treated with OP + NP both after 10 and 17 i.p. injections (Table 1). No significant body weight changes were noticed between control and study groups (data not shown).

### Hormone Plasma Levels

**Fig. 1:**
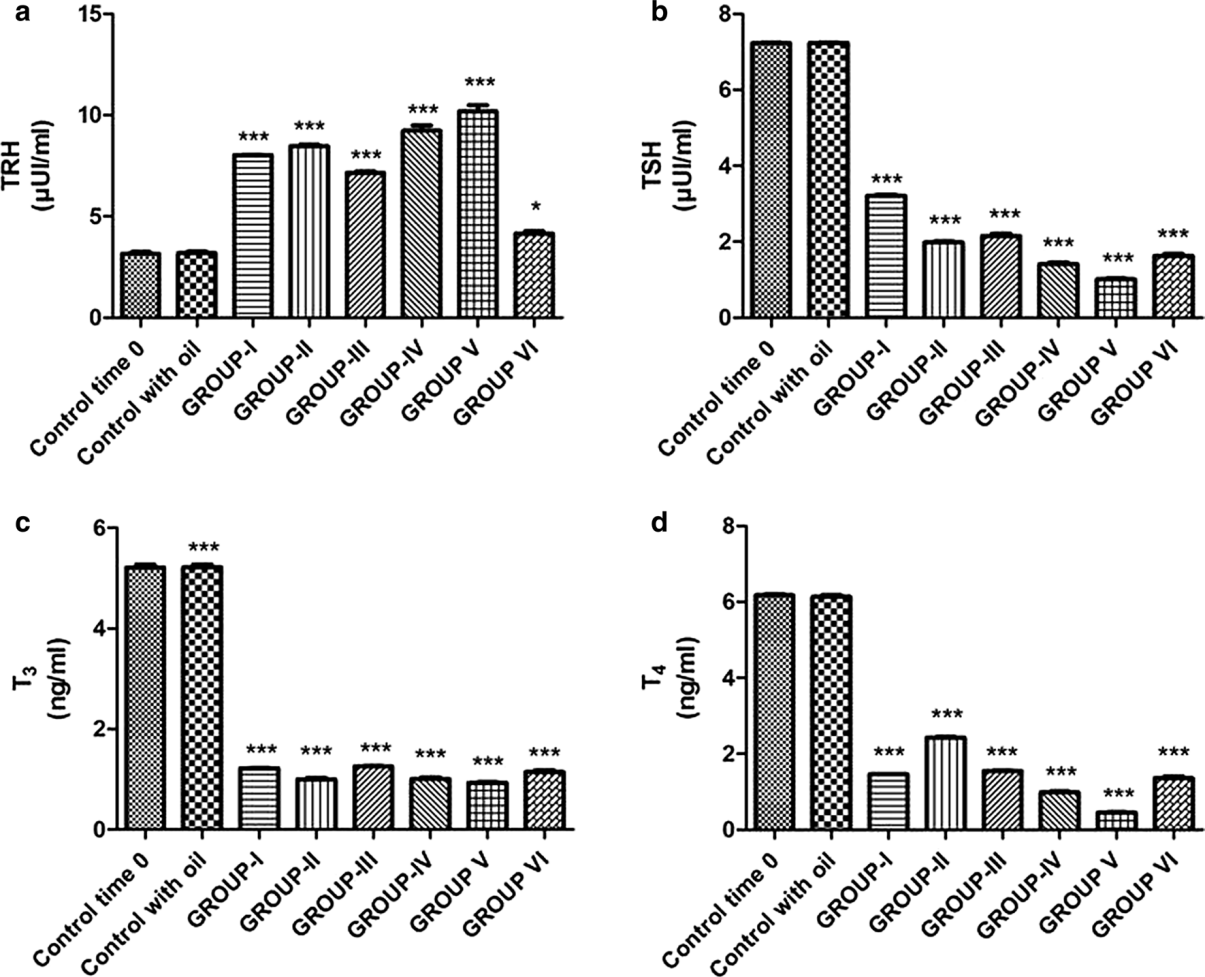
Plasma levels of TRH (**a**), TSH (**b**), T_3_(**c**), T_4_ (**d**) after OP and OP + NP treatments (*p < 0.05, ***p < 0.001, in the comparison with the control). A more detailed description in the text

Lizards treated with 12 injections of OP (Study Group I) showed an almost three-time increase in TRH level compared with control group (8.03 ± 0.40 µUI/mL vs. 3.15 ± 0.16 µUI/mL) (Table 2; Fig. 1a). In contrast, TSH plasma level decreased from 7.23 ± 0.36 µUI/mL (Control group) to 3.21 ± 0.16 µUI/mL (Group I) (Table 2; Fig. 1b). T_3_ and T_4_ values also were reduced, T_3_ passing from 5.21 ± 0.26 ng/mL (Control group) to 1.22 ± 0.06 ng/mL (Group I), and T_4_ from 6.18 ± 0.31 ng/mL (Control group) to 1.46 ± 0.07 ng/mL (Group I) (Table 2; Fig. 1c, d), which is in accordance with the decreased level of TSH.

**Table 2:** Plasma TRH, TSH, T_3_, and T_4_ levels in *P. sicula* subjected to OP and OP + NP treatments (see *Materials and Methods* section)

Treatments	TRH (µUI/mL)	TSH (µUI/mL)	T_3_ (ng/mL)	T_4_ (ng/mL)
Control time 0	3.15 ± 0.16	7.23 ± 0.04	5.21 ± 0.10	6.18 ± 0.05
Control with oil	3.19 ± 0.14	7.24 ± 0.02	5.22 ± 0.08***	6.14 ± 0.08
OP				
Group I	8.03 ± 0.05***	3.21 ± 0.06***	1.22 ± 0.02***	1.46 ± 0.02***
Group II	8.48 ± 0.11***	1.98 ± 0.05***	1.00 ± 0.05***	2.43 ± 0.03***
Group III	7.15 ± 0.15***	2.15 ± 0.10***	1.26 ± 0.01***	1.55 ± 0.02***
OP + NP				
Group IV	9.23 ± 0.46***	1.41 ± 0.07***	1.01 ± 0.05***	0.98 ± 0.05***
Group V	10.2 ± 0.51***	1.01 ± 0.05***	0.93 ± 0.04***	0.45 ± 0.02***
Group VI	4.15 ± 0.21*	1.63 ± 0.08***	1.15 ± 0.06***	1.36 ± 0.07***

Asterisks indicate statistically significant differences from the control group (*p < 0.05, ***p < 0.001)

As previously noticed, the hormonal fluctuation seemed to depend on the duration of the treatment. An even further increase in TRH plasma level (8.48 ± 0.42 µUI/mL), and decrease in TSH plasma levels (1.98 ± 0.10 µUI/mL) were observed in the specimens treated with 22 injections of OP (Study Group II) (Table 2; Fig. 1a, b). Similarly, T_3_ and T_4_ passed to the value of 1.00 ± 0.05 ng/mL and 2.43 ± 0.12 ng/mL, respectively (Study Group II) (Table 2; Fig. 1c, d).

Concentration of hormones of HPT axis was reversed in specimens treated with 22 injections of OP and sacrificed 15 days after the last injection (recovery OP group-Study Group III). In particular, a lesser increase of TRH levels was observed (7.15 ± 0.36 µUI/mL; Study Group III), but this was still significantly higher than the level recorded in the control specimens (3.15 ± 0.16 µUI/mL) (Table 2; Fig. 1a). The pituitary hormone (TSH) and the thyroid hormones (T_3_ and T_4_) also inverted the trend observed during the treatment, but similarly to TRH levels, these values remained still higher than those of the control group. In particular, increased TSH plasma levels reached 2.15 ± 0.11 µUI/mL (Table 2; Fig. 1b) and T_3_ and T_4_ were 1.26 ± 0.06 ng/mL and 1.55 ± 0.07 ng/mL, respectively (Study Group III) (Table 2; Fig. 1c, d).

Biochemical data showed that *Podarcis siculus* thyroid secretory activity was inhibited after treatment with the mixture of OctylPhenol and NonylPhenol (Table 2).

Particularly, plasma levels of the hypothalamic factor TRH increased reaching the value of 9.23 ± 0.46 µUI/mL (Study Group IV) after 10 injections of OP + NP, being three times higher than control group value (3.15 ± 0.16 µUI/mL) (Table 2; Fig. 1a). On the contrary, the plasma levels of the pituitary hormone TSH (1.41 ± 0.07 µUI/mL) (Table 2; Fig. 1b) and of the thyroid hormones T_3_ (1.01 ± 0.05 ng/mL) and T_4_ (0.98 ± 0.05 ng/mL) (Study Group IV) were significantly reduced compared with the values recorded in the control group (TSH: 7.23 ± 0.36 µUI/mL; T_3_: 5.21 ± 0.26 ng/mL; T_4_: 6.18 ± 0.31 ng/mL) (Table 2; Fig. 1c, d).

This inhibitory effect was even more prominent in the specimens treated with 17 injections of OP + NP (Study Group V) where TRH increased up to 10.2 ± 0.51 µUI/mL, and TSH, T_3_, and T_4_ decreased to 1.01 ± 0.05 µUI/mL, 0.93 ± 0.04 ng/mL, and 0.45 ± 0.02 ng/mL, respectively (Table 2; Fig. 1a–d).

Lizards treated with 17 injections of the mixture OP + NP and sacrificed 15 days after the last injection (Study Group VI-recovery OP + NP group) were characterized by a reduction in TRH plasma levels (4.15 ± 0.21 µUI/mL) and an increased TSH (1.63 ± 0.08 µUI/mL), T_3_ (1.15 ± 0.06 ng/mL), and T_4_ (1.36 ± 0.07 ng/mL) plasma levels, although these values remained higher than those recorded in the control group (Table 2; Fig. 1a–d).

### Hepatic Thyroid Hormones Content and 5-T4 ORD (type II) Monodeiodinase Activity

**Fig. 2:**
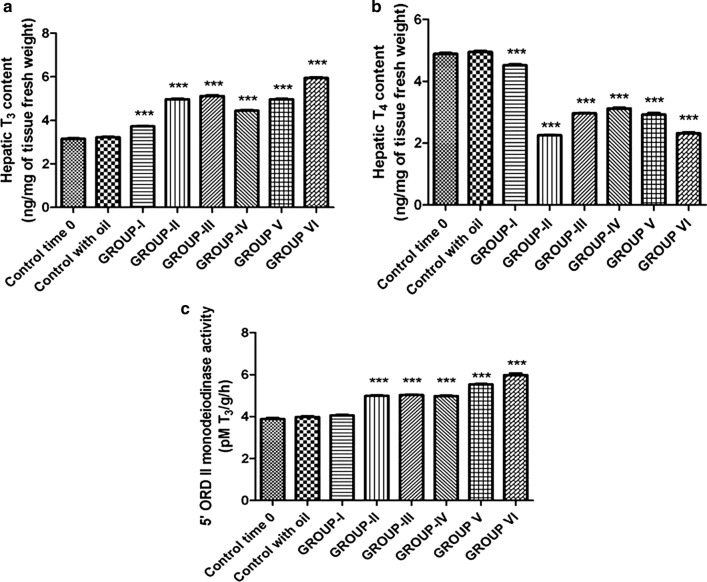
Hepatic content of T_3_ (**a**), T_4_ (**b**), 5′ORD II monodeiodinase activity (**c**) after OP and OP + NP treatments (***p < 0.001, in the comparison with the control). A more detailed description in the text

Lizards treated with OP had a higher liver T_3_ concentration and 5′ORD (type II) activity, whereas T_4_ concentration was lower than that of control lizards (Table 3; Fig. 2a–c). An increase of 5′ORD (type II) activity was observed in lizards exposed to 12 injections of OP (Study Group I) (4.06 ± 0.05 pM T_3_/g/h) and 22 injections of OP both after 24 h (4.99 ± 0.04 pM T3/g/h) (Study Group II) than that detected 15 days after the last injections (5.03 ± 0.04 pM T_3_/g/h) (Study Group III) (Table 3; Fig. 2c). A more significant increase of 5′ORD (type II) activity was observed in lizards treated with 17 injections of a mixture of OP + NP and sacrificed 15 days after the last injections (Study Group VI) (5.98 ± 0.14 pM T_3_/g/h) (Table 3; Fig. 2c).

**Table 3:** Hepatic T_3_, T_4_ content and monodeiodinase activity (type II) levels in *P. sicula* subjected to OP and OP + NP treatments (see *Materials and Methods* section)

Treatments	T_3_ (ng/mg of tissue fresh weight)	T_4_ (ng/mg of tissue fresh weight)	5′ORD II monodeiodinase activity (pM T_3_/g /h)
Control time 0	3.15 ± 0.05	4.89 ± 0.05	3.89 ± 0.08
Control with oil	3.22 ± 0.04	4.95 ± 0.06	3.98 ± 0.07
OP			
Group I	3.72 ± 0.04***	4.52 ± 0.06***	4.06 ± 0.05***
Group II	4.96 ± 0.05***	2.25 ± 0.02***	4.99 ± 0.04***
Group III	5.12 ± 0.05***	2.96 ± 0.02***	5.03 ± 0.04***
OP + NP			
Group IV	4.44 ± 0.06***	3.12 ± 0.04***	4.98 ± 0.05***
Group V	4.96 ± 0.05***	2.89 ± 0.03***	5.54 ± 0.04***
Group VI	5.95 ± 0.05***	2.32 ± 0.05***	5.98 ± 0.14***

Asterisks indicate statistically significant differences from the control group (***p < 0.001)

Hepatic T_3_ increases both in the lizards treated with OP and in those treated with OP + NP, being particularly high in animals belonging to study group VI (5.95 ± 0.05 ng/mg of tissue fresh weight**)** compared with the controls (3.15 ± 0.05 ng/mg of tissue fresh weight) (Table 3; Fig. 2a). On the contrary, the hepatic contents of T_4_ decreased in all groups treated with OP and OP + NP, becoming especially low in animals treated with 17 injections of OP + NP and sacrificed 15 days after the last injection (Group VI- recovery OP + NP group) (2.32 ± 0.05 ng/mg of tissue fresh weight) (Table 3; Fig. 2b).

### Thyroid Gland Histology After Treatments

**Fig. 3:**
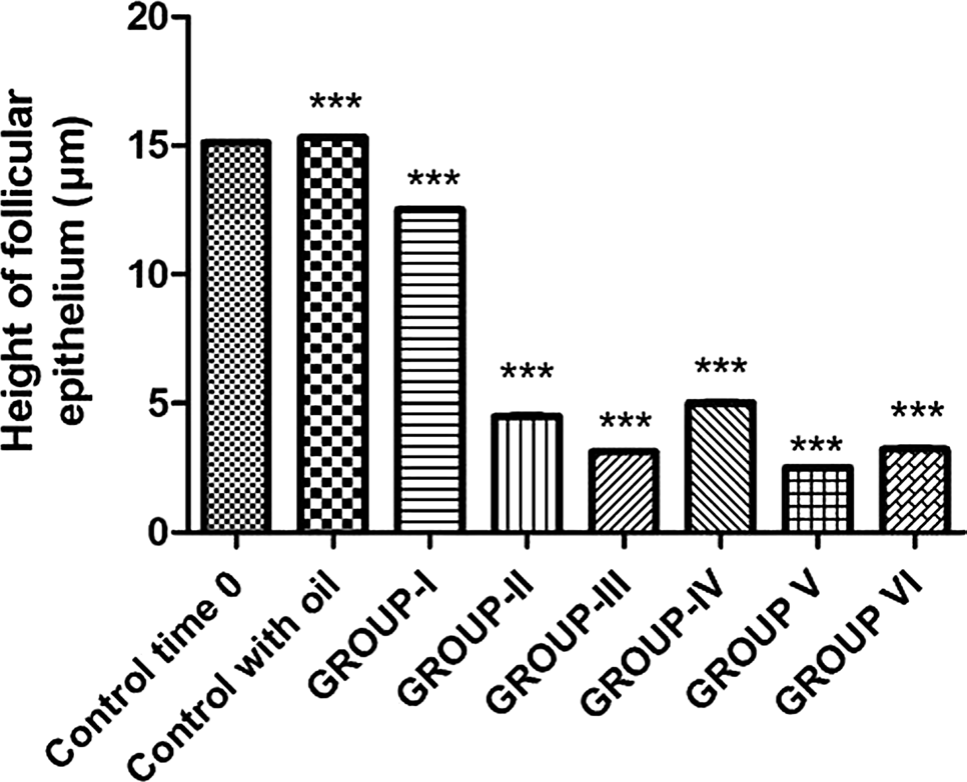
Height of follicular epithelium after OP and OP + NP treatments (***p < 0.001, in the comparison with the control). A more detailed description in the text

**Fig. 4:**
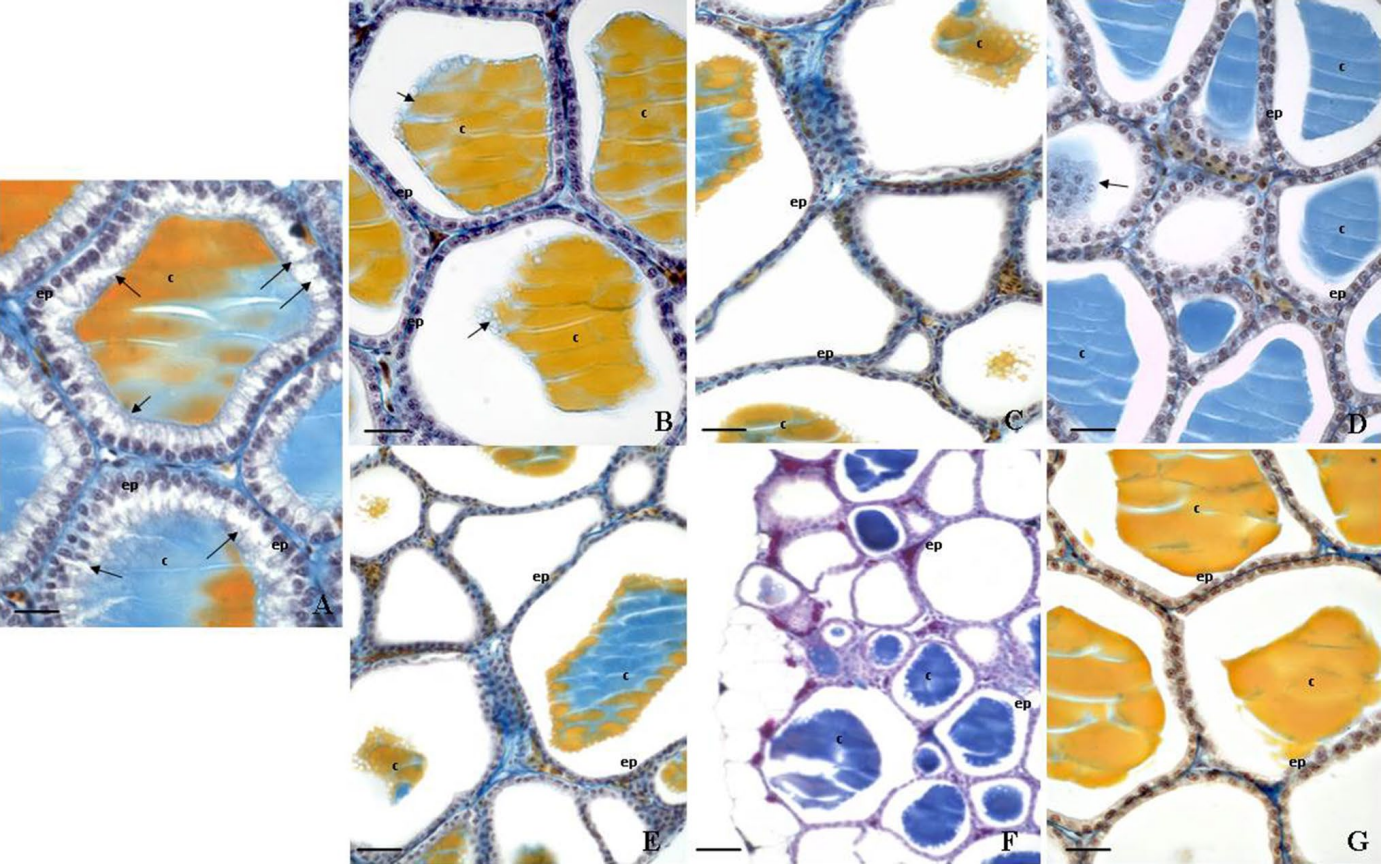
Thyroid gland of lizard’s *P. siculus* (stain Galgano I); Scale bar: 20 μm. **a** Control lizard: the cuboidal follicular epithelial cells (ep), the colloid (c), and the reabsorption vacuoles (arrow) are shown. **b** Lizards treated with OP for 12 days and sacrificed 24 h after the last injection: the follicular epithelium (ep) is lower than in control animals. **c** Lizards treated with OP for 22 days and sacrificed 24 h after the last injection: the follicular epithelium (ep) is very low and the decrease of colloids (c) in follicles is very evident compared with untreated animals. **d** Lizards treated with OP for 22 days and sacrificed 15 days after the last injection (recovery OP group): the follicular epithelium (ep) is lower than normal, but the colloid (c) is present in the follicles. **e** Lizards treated with OP + NP for 10 days and sacrificed 24 h after the last injection: the follicular epithelium (ep) is lower than normal and no reabsorbing vacuoles are visible in the colloid (c). **f** Lizards treated with OP + NP for 17 days and sacrificed 24 h after the last injection: the follicular epithelium (ep) is very low compared with normal epithelium, and no reabsorbing vacuoles can be seen in the colloid (c). **g** Lizards treated with OP + NP for 17 days and sacrificed 15 days after the last injection (recovery OP + NP group); the follicular epithelium (ep) is lower than normal epithelium, but the colloid (c) is present in the follicles

The thyroid gland transversely crosses the middle of the trachea in *Podarcis siculus* specimens, looking like a ribbon-shaped structure consisting of follicles that are connected to each other by an interfollicular connective tissue that holds blood vessels. The gland is wrapped in a capsule of superficial connective tissue that branches out and forms a network that surrounds the follicles. Each follicle is enveloped in a high cuboidal epithelium (15.1 ± 0.02 µm) (Table 4; Fig. 3), formed by thyrocytes and containing a medium-sized colloidal mass (Fig. 4a).

**Table 4:** Variations of epithelium height of the follicular cells of the thyroid gland in *P. sicula* subjected to OP and OP + NP treatments (see *Materials and Methods* section)

Treatments	Height of follicular epithelium (µm)
Control time 0	15.1 ± 0.02
Control with oil	15.3 ± 0.05
OP	
Group I	12.5 ± 0.03***
Group II	4.51 ± 0.04***
Group III	3.12 ± 0.03***
OP + NP	
Group IV	5.01 ± 0.03***
Group V	2.50 ± 0.02***
Group VI	3.23 ± 0.05***

Asterisks indicate statistically significant differences from the control Group (***p < 0.001)

The thyroid gland of lizards treated with 12 injections of OP appeared richly vascularized with a medium follicular epithelium (12.5 ± 0.03 µm) (Table 4; Fig. 3); thyrocytes had still a cubic shape and colloid showed numerous reabsorption vacuoles (Fig. 4b). After 22 injections of OP, lizards showed a thyroid gland with a lower follicular epithelium (4.51 ± 0.04 µm) (Table 4; Fig. 3) and a retracted colloid without reabsorption vacuoles (Fig. 4c). Morphologically, in specimens treated with 22 injection of OP and sacrificed 15 days after the last injections (recovery OP group), there was no histological sign of recovery, which is in contrast to what was observed from a hormonal point of view (Fig. 4d).

Morphological analysis of the thyroid gland in specimens treated with 10 injections of the mixture OP + NP showed a slight reduction in the height of the follicular epithelium and a partial retroaction of the colloid (Fig. 4e), which is in accordance with hormonal dosages results. Overlapping results were observed in the group treated with 17 injections of the mixture OP + NP (Fig. 4f). The follicular epithelium was lower than normal (2.5 ± 0.02 µm) (Table 4; Fig. 3), and the nuclei of the thyrocytes were small and elongated with dense chromatin and a greatly reduced cytoplasm. The colloid was retracted with few reabsorption vacuoles. The thyroid gland showed very evident signs of a poor functional activity. In the recovery OP + NP group, the histological evaluation did not show any signs of recovery of the organism (Fig. 4g).

## Discussion

The release, accumulation, and fate of numerous pollutants in the environment is attracting a lot of attention for its potential negative impact on human health. Several environmental pollutants are classified as endocrine-disrupting chemicals (EDCs) for their potential to disrupt the endocrine system. Thyroid can be a target of different EDCs, which can impair its physiology at different levels, including its central regulatory system in the hypothalamus and pituitary axis, thyroid hormone production and transfer, as well as hormone function, metabolism, and bioavailability (Calsolaro et al. 2017; Mughal et al. 2018).

In the lizard *Podarcis siculus*, thyroid hormones (THs) production is primarily regulated by two components: “central control” and “peripheral control” (Sciarrillo et al. 2000). Under the central control, the thyroid-stimulating hormone (TSH), a glycoprotein secreted by the anterior pituitary gland, regulates the synthesis and release of THs by the thyroid follicles. In lizards, 3,3′,5,5′-L-thyroxine (T_4_) is the predominant thyroid follicle secretion (Sciarrillo et al. 2000); it has strong negative feedback effects on TSH levels. T_4_ is transported to the peripheral tissues (e.g., liver) via the circulatory system and is either converted to the more bioactive 3,3′,5-triiodo-L-thyronine (T_3_) or to the inactive degradation products by iodothyronine deiodinase (type II, D2) under peripheral regulation. The primary control of T_3_ levels is known to occur mainly in peripheral tissues (Sciarrillo et al. 2000). THs exert their physiological effects mainly by interacting with the nuclear thyroid receptors (TRs), which belong to a large super-family of ligand-induced transcription factors (Virgilio et al. 2004).

To date, few investigations have been conducted about the effects of alkylphenols on the thyroid gland. Therefore, in this study we have evaluated the impact of OP as such or in combination with NP on the thyroid gland of the lizards *Podarcis siculus* in vivo. Our results indicate that treatment with both compounds cause structural and functional alterations in the thyroid gland of the lizards. In particular, OP and OP + NP treatments caused a strong time- and dose-dependent inhibition of the functionality of the gland, which is in line with what we previously reported for NP treatment (Sciarrillo et al. 2010).

Functionally, OP and OP + NP treatments might cause hypothyroidism as they determine a secretory blockage in the pituitary gland. This conclusion emerged from the analysis of biochemical data that showed an increase in TRH levels and a decrease in the hormones TSH, T_3_, and T_4_ following both treatments.

Structurally, lizards exposed to OP and OP + NP showed a reduced height of the follicular epithelium and a retracted colloid with fewer reabsorption vacuoles. Follicular epithelium height and colloid are indices of secretory activity of the gland (Movahedinia et al. 2018) as well as useful parameters for the observation of EDC morphological effects on the thyroid. Moreover, epithelial cell height is a histological method of thyroid gland assessment as it is considered to be roughly proportional to the degree of response to thyroid-stimulating hormone (TSH) (Moccia et al. 1981). In this study, OP and OP + NP induced a decrease in the height of thyroid follicular cells, follicular cell hyperplasia, shrunken follicular epithelial cells, and decreased cytoplasm quantity in the thyroid gland. These observation suggest that EDCs could influence the microscopic structures of the thyroid gland. In addition, it is possible to speculate that the HPT axis may be targeted by AlkylPhenolic compounds, such as OP, alone or in combination with NP.

Our results confirm the inverse association between APs doses and circulating levels of thyroid hormones in *Podarcis siculus* lizards. The thyroid function is regulated by sensitive feedback mechanisms of circulating thyroid hormones at the hypothalamic (TRH) and pituitary levels (TSH). The appropriate response of the feedback would result in declined/reduced TSH levels, which might result in compensatory hypoplasia of thyroid tissue. Histopathological changes that we observed in all treated animals suggest the possible effects of APs on feedback mechanisms of Hypothalamic-Pituitary-Thyroid (HPT) axis (Santos-Silva et al. 2018; Sheikh 2020; Xie et al. 2019).

Despite advances in analytical methods to study these chemicals in biological tissues, the identification of reliable markers to measure the effects of APs on thyroid function, the exact way, and/or mechanisms of action remains still difficult. APs may interfere with TH homeostasis in different ways. Many of the physiological functions of tissue cells (i.e., liver) depend on the dynamic regulation of THs and therefore the iodothyronine deiodinase is critical to this regulation. Hepatic 5′ORD (Type II) activity in all treated groups was significantly higher than in the control group, along with a decreased T_4_ and an increased T_3_ contents. Based on these observations, we hypothesize that reduction of liver T_4_ content is dependent on the down-regulation of 5′ORD (Type II) after exposure to APs. These results indicate that APs could induce an abnormal thyroid function by influencing levels of deiodinases in peripheral tissues (i.e., liver) and TRs. Environmental APs might enhance the metabolic rate of TH in vivo by inhibiting binding of THs to TRs, thus causing a decrease in TH activity and an increase in iodothyronine deiodinase activity. Inhibition of TH-binding by OP might damage the TRH–TSH–THs regulatory pathway. In addition, APs might accelerate the TH metabolic rate by enhancing the activity of iodothyronine deiodinase, leading to a decrease in T_3_ and T_4_ activity (He et al. 2020).

APs might influence TH activity via the regulation of multiple targets within the complex regulatory network of TH metabolism and activity, including TRs binding and activation. This mechanism mediates gene regulation in response to T_3_ deiodinase, which catalyzes deiodination of T_4_ to be converted to the biologically active T_3_ form, and the Hypothalamus–Pituitary–Thyroid axis, which contains the TRH–TSH–THs negative feedback (Street et al. 2018).

## Conclusions

Our results suggest that EDCs and in particular APs interfere with thyroid function in *P. siculus* at different levels, including the central regulatory system in the hypothalamus and pituitary, thyroid hormone production at the thyroid gland, thyroid hormone transfer, as well as hormone bioavailability, function, and metabolism in peripheral organs.
